# Genome-wide identification and characterization of bZIP gene family and cloning of candidate genes for anthocyanin biosynthesis in pomegranate (*Punica granatum*)

**DOI:** 10.1186/s12870-022-03560-6

**Published:** 2022-04-04

**Authors:** Sha Wang, Xinhui Zhang, Bianbian Li, Xueqing Zhao, Yu Shen, Zhaohe Yuan

**Affiliations:** 1 Co-Innovation Center for Sustainable Forestry in Southern China, Nanjing Forestry University, Nanjing, 210037 China; 2 College of Forestry, Nanjing Forestry University, Nanjing, 210037 China

**Keywords:** Pomegranate, bZIP transcription factor family, Anthocyanin, Gene cloning, Subcellular localization

## Abstract

**Background:**

The basic leucine zipper (bZIP) transcription factor is one of the most abundant and conserved gene families in eukaryotes. In addition to participating in plant development and growth, bZIP transcription factors play crucial roles in various abiotic stress responses and anthocyanin accumulation. Up to now, analysis of bZIP gene family members in pomegranate (*Punica granatum*) has not been reported. Three published pomegranate genome sequences provide valuable resources for further gene function analysis.

**Results:**

Using bioinformatics analysis, 65 PgbZIPs were identified and analyzed from the ‘Taishanhong’ pomegranate genome. We divided them into 13 groups (A, B, C, D, E, F, G, H, I, J, K, M, and S) according to the phylogenetic relationship with those of *Arabidopsis*, each containing a different number of genes. The regularity of exon/intron number and distribution was consistent with the classification of groups in the evolutionary tree. Transcriptome analysis of different tissues showed that members of the PgbZIP gene family were differentially expressed in different developmental stages and tissues of pomegranate. Among them, we selected *PgbZIP16* and *PgbZIP34* as candidate genes which affect anthocyanin accumulation. The full-length CDS region of *PgbZIP16* and *PgbZIP34* were cloned from pomegranate petals by homologous cloning technique, encoding 170 and 174 amino acids, which were 510 bp and 522 bp, respectively. Subcellular localization assays suggested that both PgbZIP16 and PgbZIP34 were nucleus-localized. Real-time quantitative PCR (qPCR) was used to explore the expression of *PgbZIP16* and *PgbZIP34* in the petals of three kinds of ornamental pomegranates at the full flowering stage. The results demonstrated that the expression of *PgbZIP16* in red petals was 5.83 times of that in white petals, while *PgbZIP34* was 3.9 times. The results of transient expression in tobacco showed that consistent trends were observed in anthocyanin concentration and expression levels of related genes, which both increased and then decreased. Both *PgbZIP16* and *PgbZIP34* could promote anthocyanin accumulation in tobacco leaves. We obtained transgenic strains overexpressing *PgbZIP16*, and the histochemical staining for GUS activity showed that overexpressed *PgbZIP16* seedlings were expressed in the stem. Transgenic experiments indicated that overexpression of *PgbZIP16* significantly upregulated *UF3GT*, *ANS* and *DFR* genes in *Arabidopsis* and enhanced anthocyanin accumulation.

**Conclusions:**

The whole genome identification, gene structure, phylogeny, gene cloning, subcellular location and functional verification of the pomegranate bZIP gene family provide a theoretical foundation for the functional study of the PgbZIP gene family and candidate genes for anthocyanin biosynthesis.

**Supplementary Information:**

The online version contains supplementary material available at 10.1186/s12870-022-03560-6.

## Background

Anthocyanins belonging to flavonoids are water-soluble pigments providing various color for plants, especially in fruits and flowers [1]. Anthocyanin accumulation in plants is usually to resist abiotic stress, such as drought, ultraviolet radiation, hormone and low temperature [2–5]. Recently, anthocyanins had potential health benefits that they deserved increasing attention. It has presented that daily intake of natural foods rich in anthocyanin has a potent protective effect on the human body. Moreover, it can play a role in preventing cardiovascular disease and obesity [6–9]. In many plants, it has been proposed that the anthocyanin biosynthesis pathway is mainly regulated by R2R3-MYB, bHLH and WD40 repeats factors to control their downstream structural genes [10]. While one another transcription factor bZIP is found to promote anthocyanin synthesis in combination with MYBs to increase their expression [11].

Transcription factors (TFs) are the most important part of the plant growth and development regulatory network, which activate or inhibit genes expression by combining with specific promoter sequences. bZIP transcription factors regulate many plant processes through the interaction of DNA binding motifs, transcriptional activation motifs, nuclear localization signals and oligomerization sites [2]. Many TFs can be classified into different gene families according to their conserved domains. Presently, at least 64 families of transcription factors have been identified in the plants [12]. As one of the most abundant and conserved gene families in eukaryotes, the basic leucine zipper motif (bZIP) gene family has an integral role in growth development and abiotic stress responses in plants [13]. The conserved bZIP domain has about 40 ~ 80 amino acid residues, which includes two parts, a highly conserved DNA-binding basic composed of 20 amino acids and a relatively diversified leucine zipper region [14]. The basic amino acid region is located at the C-terminal region and through a fixed N-x7-R/K structure for sequence-specific DNA binding. The leucine zipper region located at the N-terminal region, which consists of several heptapeptide repeats or hydrophobic amino acid residues, such as methionine, isoleucine, valine, etc. This domain main function by forming dimers through the leucine zipper domain [15, 16].

The bZIP transcription factor family has been comprehensively identified in several plants, such as 78, 58, 69, 55, 89, 114, 45, 45 in *Arabidopsis* [17, 18], maize (*Zea mays*) [19], tomato (*Solanum lycopersicum*) [20], grape (*Vitis vinifera*) [21], rice (*Oryza sativa*) [13], apple (*Malus domestica*) [22], poplar (*Populus simonii*) [23], and Chinese jujube (*Ziziphus jujuba*) [24], respectively. The *Arabidopsis* bZIP gene family consists of 78 members divided into 13 groups (groups A-K, M and S) [18]. Currently, a large number of *bZIP* genes have been found to play important roles in the processes of plant growth and development, such as seed maturation and germination [25], flower development [26], vascular development [27] and embryogenesis [28]. For example, the *AtbZIP11* affects plant root development by linking low-energy signals to auxin-mediated control of primary root growth [29]. Overexpression of the *ZmbZIP4* in maize can also lead to an increase in the number of lateral roots, longer primary roots and improved plant roots [30]. In addition, the *bZIP* genes also play an important role in plant biotic and abiotic stress [31–33]. In wheat, *TabZIP15* promotes the combination of ABF/AREB and ABRE (ABA response element) cis-acting elements through the expression of ABF/AREB to induce downstream target gene expression responding to plant salt and drought stress [34]. Similar results were observed for the *GsbZIP67* gene in Alfalfa (*Medicago sativa*), overexpression of *GsbZIP67* promoted the growth of plant roots and shoots and changed the physiological indicators of transgenic plants under bicarbonate salt-alkali stress [32].

Interestingly, a large number of studies have shown that some *bZIP* genes are involved in plant anthocyanin biosynthetic pathway [35]. ELONGATED HYPOCOTYL5 (HY5), one member of the bZIP gene family, was activated in a light-dependent manner to promote pigment accumulation. *HY5* could directly bind to G-box or ACE-box of MYB factors, including PRODUCTION OF ANTHOCYANIN PIGMENT1 (*PAP1*), PRODUCTION OF FLAVONOL GLYCOSIDES (*MYB12* and *MYB111*), and *MYB-like Domain* (*MYBD*) to promote their gene expression [36–39]. Besides *MYBs*, *HY5* co-regulate with *PIF3* the expression of anthocyanin biosynthesis structure genes [35]. Moreover, it has proposed that the overexpression *bZIP* gene *MdHY5* in apple callus induce anthocyanin accumulation by upregulating *MdMYB10* expression and its downstream genes [40]. Overexpression of *CRY1a* could increase accumulation of anthocyanin in tomato and *SlHY5* silencing could decrease *CRY1a*-induced anthocyanin accumulation [41]. In addition, *HY5* positively regulates the cold responses through activation of anthocyanin biosynthesis genes such as chalcone synthase (*CHI*) and chalcone isomerase (*CHS*) [42]. Under the induction of abscisic acid (ABA), *MdbZIP44* (*HY5*) positively regulates the anthocyanin accumulation by enhancing the interaction between *MdMYB1* and its downstream target genes [43].

Pomegranate (*Punica granatum*) belongs to the Lythraceae family. It is one of the important characteristic economic forest species in the world. It has achieved considerable attention due to its high antioxidant activity, rich color in peel and aril (the edible part of pomegranate), nutritious, active pharmaceutical ingredients and anthocyanin [44, 45]. Pomegranate has fruit and flower pomegranate, its attractive appearance, the long forefronts of flowering, and it is gradually being used in landscaping. Comprehensive analysis, pomegranate has strong health functions, high ornamental value and ecological and economic profitability, and has great development prospects [46]. In recent years, most scholars have sequenced and assembled the genomes of different pomegranate varieties, and obtained high-quality genome maps, such as ‘Dabenzi’ [47], ‘Taishanhong’ [44] and ‘Tunisia’ soft-seed pomegranate [48], which provide an important molecular biology basis for pomegranate genetic improvement research.

In this study, we use bioinformatics methods to identify the members of the pomegranate bZIP transcription factor family members, and analyze the physical and chemical properties, conserved domains, evolutionary relationships, cis-acting elements, tissue and organ expression of transcription factors. At the same time, two candidate genes related to anthocyanin synthesis were identified in pomegranate for the first time, and the gene cloning, subcellular location and differential expression of flower with different colors were analyzed. These results provide a reference for studying the expression of pomegranate bZIP gene family during the growth and development of pomegranate and biotic and abiotic stress, and provide a basis for further elucidating the formation mechanism of pomegranate flower color.

## Results

### Identification and characterization of the bZIP transcription factor family in pomegranate

In this study, we identified 65 gene family members from the whole genome of ‘Taishanhong’ pomegranate. For subsequent analysis, they renamed them according to scaffold (Table 1). The physicochemical properties of bZIP transcription factors were analyzed by ExPASy online-tool. The results showed that the molecular weight of pomegranate bZIP family proteins ranged from 14,803.81 to 380,571.64 Da, the theoretical isoelectric point ranged from 4.74 to 9.91, and the protein lengths ranged from 128 to 1543 aa, with the shortest being 128 aa (PgbZIP11) and the longest being 1543 aa (PgbZIP8). These results provide a theoretical basis for further purification, activity and function studies of PgbZIP protiens. The subcellular location prediction of each member indicated that all members of the bZIP gene family are expressed in the nucleus.

**Table 1:** The identified *PgbZIP* genes and their related information

Gene Name	Gene ID	Location	Group	CDS	AA	MW(Da)	pI	Subcelluar Localization
*PgbZIP1*	Pg000486.1	scaffold1:692075:692518	S	443	147	16,488.75	7.86	Nucleus
*PgbZIP2*	Pg000487.1	scaffold1:695206:695652	S	447	149	36,053.89	5.18	Nucleus
*PgbZIP3*	Pg000951.1	scaffold1:2591499:2593093	I	729	243	59,393.95	5.12	Nucleus
*PgbZIP4*	Pg011031.1	scaffold2:1782851:1795679	D	2265	755	184,985.25	4.88	Nucleus
*PgbZIP5*	Pg011379.1	scaffold2:3151711:3152349	S	639	213	54,055.25	5.09	Nucleus
*PgbZIP6*	Pg010908.1	scaffold2:3418553:3421108	C	1320	440	47,485.28	7.75	Nucleus
*PgbZIP7*	Pg016213.1	scaffold3:1034859:1035461	S	600	200	22,636.22	5.59	Nucleus
*PgbZIP8*	Pg019746.1	scaffold4:1634806:1637852	I	1041	347	87,468.71	4.97	Nucleus
*PgbZIP9*	Pg019532.1	scaffold4:1723642:1725546	A	834	278	30,788.86	9.29	Nucleus
*PgbZIP10*	Pg019474.1	scaffold4:2417973:2420458	A	834	278	29,691.17	5.91	Nucleus
*PgbZIP11*	Pg019929.1	scaffold4:3861901:3862287	S	384	128	14,981.66	6.91	Nucleus
*PgbZIP12*	Pg022634.1	scaffold5:1188977:1190472	A	780	260	28,329.43	8.35	Nucleus
*PgbZIP13*	Pg022422.1	scaffold5:1764422:1764892	S	471	157	38,400.71	5.17	Nucleus
*PgbZIP14*	Pg022742.1	scaffold5:2775118:2777326	A	1311	437	46,593.41	9.68	Nucleus
*PgbZIP15*	Pg022303.1	scaffold5:3712021:3712449	S	426	142	16,587.97	9.42	Nucleus
*PgbZIP16*	Pg024592.1	scaffold6:2540285:2543069	H	507	169	18,466.50	9.91	Nucleus
*PgbZIP17*	Pg026584.1	scaffold7:690047:692762	A	1038	346	38,062.41	8.42	Nucleus
*PgbZIP18*	Pg026564.1	scaffold7:944817:948069	D	1164	388	95,110.83	5.03	Nucleus
*PgbZIP19*	Pg026477.1	scaffold7:2126264:2129463	J	1554	518	125,872.84	4.98	Nucleus
*PgbZIP20*	Pg026888.1	scaffold7:2982554:2984794	A	1245	415	44,938.52	9.49	Nucleus
*PgbZIP21*	Pg001581.1	scaffold10:3315235:3317319	E	1125	375	92,957.96	4.98	Nucleus
*PgbZIP22*	Pg002913.1	scaffold11:2030336:2032657	A	1266	422	45,882.25	8.87	Nucleus
*PgbZIP23*	Pg003837.1	scaffold12:3213559:3215308	E	891	297	32,957.73	5.79	Nucleus
*PgbZIP24*	Pg005395.1	scaffold13:113807:114247	S	438	146	16,629.94	6.37	Nucleus
*PgbZIP25*	Pg005194.1	scaffold13:3081012:3081581	S	567	189	21,419.06	8.93	Nucleus
*PgbZIP26*	Pg008236.1	scaffold16:613569:614306	F	738	246	60,324.09	5.10	Nucleus
*PgbZIP27*	Pg008260.1	scaffold16:920825:924942	D	1296	432	106,682.40	5.00	Nucleus
*PgbZIP28*	Pg008051.1	scaffold16:1996563:1999431	D	1254	418	102,298.97	5.01	Nucleus
*PgbZIP29*	Pg008941.1	scaffold17:183002:184828	A	813	271	28,884.20	9.44	Nucleus
*PgbZIP30*	Pg008855.1	scaffold17:988420:990879	G	1035	345	36,244.38	5.39	Nucleus
*PgbZIP31*	Pg009560.1	scaffold18:76414:78344	E	927	309	76,112.58	5.08	Nucleus
*PgbZIP32*	Pg009379.1	scaffold18:2446066:2448483	I	924	308	75,878.49	5.04	Nucleus
*PgbZIP33*	Pg010362.1	scaffold19:3431036:3432620	A	1128	376	41,024.31	8.95	Nucleus
*PgbZIP34*	Pg012358.1	scaffold21:2286741:2288172	H	519	173	19,322.86	9.89	Nucleus
*PgbZIP35*	Pg013506.1	scaffold22:1812013:1819851	B	4629	1543	380,571.64	4.74	Nucleus
*PgbZIP36*	Pg013360.1	scaffold22:5624189:5627104	I	1074	358	88,826.51	4.98	Nucleus
*PgbZIP37*	Pg013743.1	scaffold23:1468781:1470885	I	1299	433	107,204.34	4.98	Nucleus
*PgbZIP38*	Pg014054.1	scaffold24:1165026:1166900	D	705	235	57,216.90	5.14	Nucleus
*PgbZIP39*	Pg014221.1	scaffold24:1205470:1206418	A	807	269	30,034.36	9.23	Nucleus
*PgbZIP40*	Pg015819.1	scaffold29:475717:476172	S	453	151	16,520.56	9.50	Nucleus
*PgbZIP41*	Pg015844.1	scaffold29:999707:1002603	I	1806	602	148,814.05	4.92	Nucleus
*PgbZIP42*	Pg015870.1	scaffold29:1365294:1366040	F	747	249	59,751.28	5.14	Nucleus
*PgbZIP43*	Pg016758.1	scaffold30:1040570:1041935	A	798	266	29,406.84	9.23	Nucleus
*PgbZIP44*	Pg017636.1	scaffold33:350792:351202	S	411	137	32,817.67	5.23	Nucleus
*PgbZIP45*	Pg017872.1	scaffold34:149123:150849	F	870	290	68,888.04	5.12	Nucleus
*PgbZIP46*	Pg017805.1	scaffold34:1334693:1335175	S	480	160	17,740.69	6.31	Nucleus
*PgbZIP47*	Pg018175.1	scaffold35:221629:223223	K	822	274	66,700.39	5.10	Nucleus
*PgbZIP48*	Pg018974.1	scaffold38:374554:377270	C	1221	407	43,936.74	5.58	Nucleus
*PgbZIP49*	Pg019276.1	scaffold39:1591489:1593020	K	909	303	74,386.34	5.07	Nucleus
*PgbZIP50*	Pg020198.1	scaffold40:873396:873791	S	393	131	14,803.81	9.35	Nucleus
*PgbZIP51*	Pg020064.1	scaffold40:1476711:1477762	A	657	219	24,176.26	9.47	Nucleus
*PgbZIP52*	Pg021901.1	scaffold48:629216:639124	D	1371	457	112,445.22	5.00	Nucleus
*PgbZIP53*	Pg023016.1	scaffold50:634939:635538	S	597	199	22,700.04	5.60	Nucleus
*PgbZIP54*	Pg023400.1	scaffold52:433306:436854	G	1194	398	42,055.23	6.26	Nucleus
*PgbZIP55*	Pg023806.1	scaffold54:1143344:1149748	M	888	296	73,776.05	5.05	Nucleus
*PgbZIP56*	Pg023869.1	scaffold55:84392:86720	G	1053	351	37,924.99	5.56	Nucleus
*PgbZIP57*	Pg025024.1	scaffold60:329234:333156	D	1542	514	125,729.20	4.97	Nucleus
*PgbZIP58*	Pg028547.1	scaffold80:538801:545326	D	1446	482	119,119.95	4.94	Nucleus
*PgbZIP59*	Pg028644.1	scaffold80:650755:652351	J	894	298	73,357.03	5.07	Nucleus
*PgbZIP60*	Pg029370.1	scaffold87:425556:427793	E	975	325	80,691.55	5.01	Nucleus
*PgbZIP61*	Pg002089.1	scaffold105:550189:557112	D	1461	487	119,232.03	4.96	Nucleus
*PgbZIP62*	Pg003440.1	scaffold114:422966:423556	S	591	197	49,650.00	5.11	Nucleus
*PgbZIP63*	Pg004996.1	scaffold128:119987:121887	A	978	326	35,872.31	8.98	Nucleus
*PgbZIP64*	Pg011906.1	scaffold201:109649:114841	C	1230	410	44,481.01	5.41	Nucleus
*PgbZIP65*	Pg012452.1	scaffold211:113511:113990	S	477	159	17,460.66	6.60	Nucleus

### Phylogenetic tree and bZIP conservative domain analysis

**Fig. 1:**
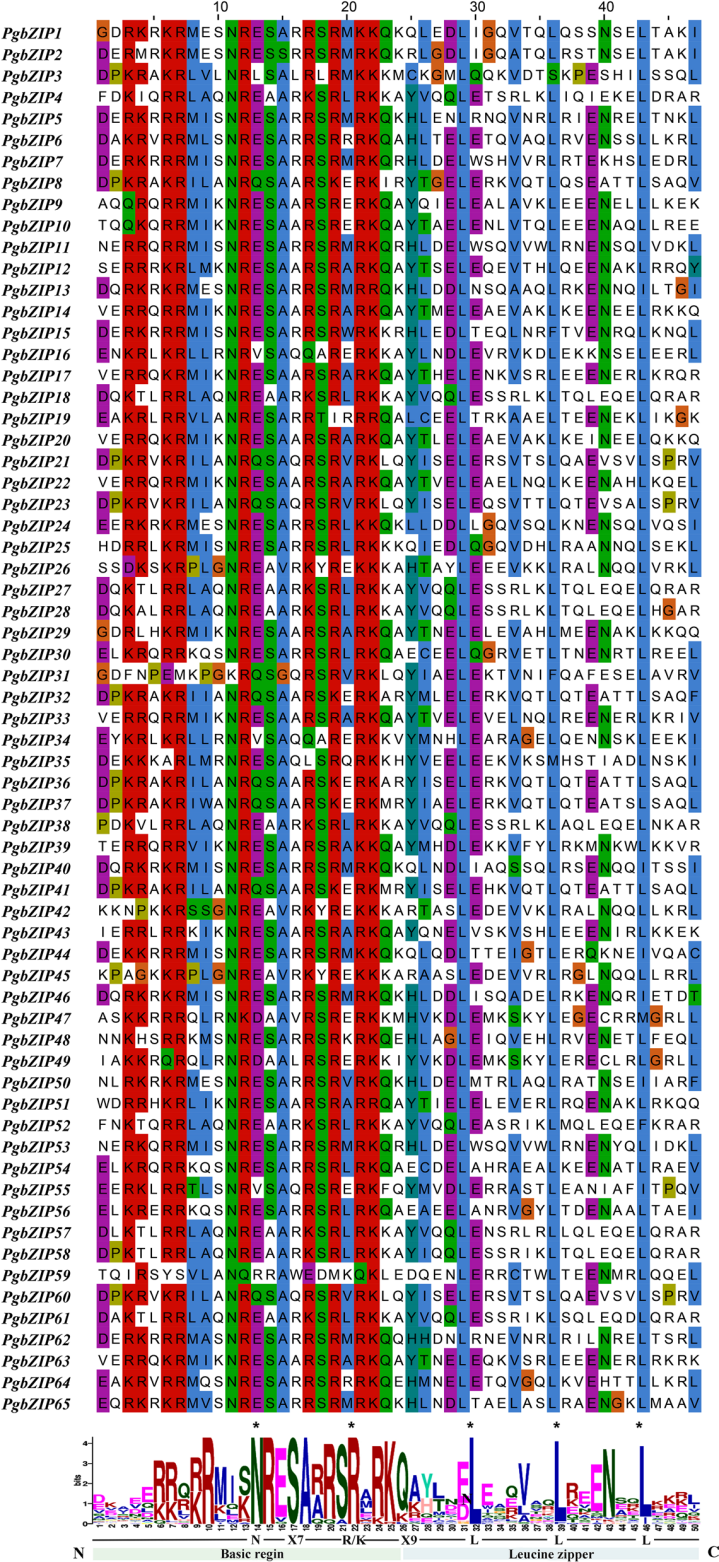
Visualization of multiple sequence alignment of the pomegranate bZIP family DNA binding domains. The total height of the letter piles at each position indicates the conservation of the sequence at that position (measured in bits). The height of a single letter in the letter piles represents the relative frequency of the corresponding amino acid at that position

The amino acid positions of the conserved structural domains of bZIP were visualized by multiple sequence alignment of the protein sequences of 65 bZIP family members of pomegranate (Fig. 1). The results showed that the core conserved structural domain of the PgbZIPs protein had an average length of 50 aa. The bZIP structural domain consists of a basic region and a leucine zipper. the basic region was located at the the C-terminus and contains a fixed N-X7-R/K motif bound to a specific DNA sequence, while the leucine zipper region was located at the N-terminal end and consists of several repetitive heptapeptide or hydrophobic amino acid residues. The highly conserved leucine residues were sometimes replaced by isoleucine, methionine, valine, etc. Our results are consistent with previous study in *Arabidopsis* [18]*.*

**Fig. 2:**
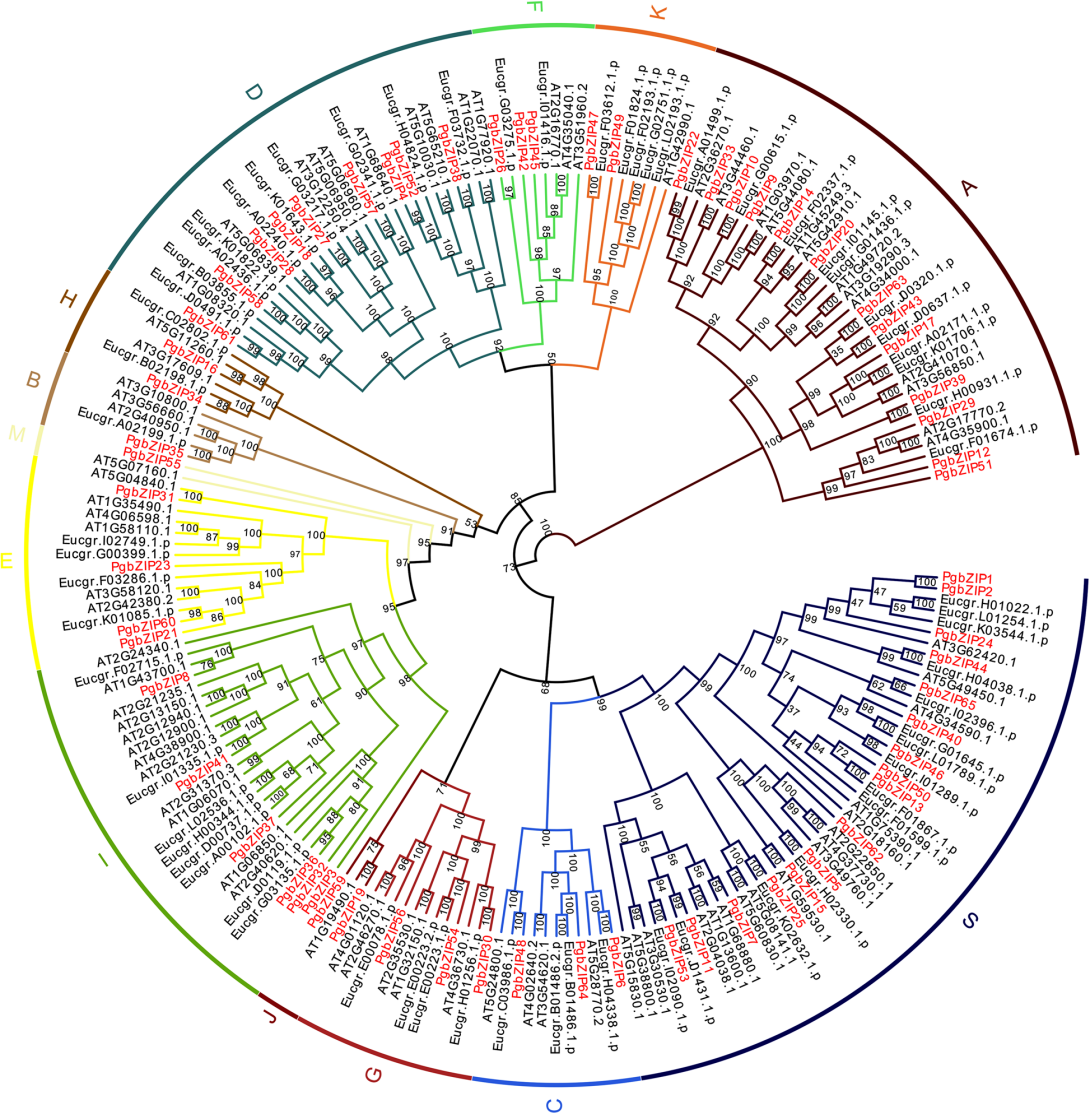
The unrooted phylogenetic tree of *P. granatum, E. grandis *and *Arabidopsis* bZIP proteins. The dendrogram was drew by MEGA7 with the Maximum Likelihood method and JTT + G + F model. Different groups are marked with different colors. The groups were named with letters representing some of their important members (A for ABF/AREB/ABI5, C for CPRF2-like, G for GBF, H for HY5), protein size (B for big, S for small), or alphabetically

To explore the homologous evolutionary relationships and classification of the bZIP family, we constructed a phylogenetic tree using the bZIP members of pomegranate, *Eucalyptus megacephalus* and *Arabidopsis*. As shown in Fig. 2, a deep clustering analysis of the entire evolutionary tree was performed with reference to the evolutionary relationship and naming rules of the *Arabidopsis* bZIP genes. The pomegranate bZIP gene family was divided into 13 groups (A, B, C, D, E, F, G, H, I, J, K, M, and S). These 13 groups differed greatly in size. Two of the groups have only one member, namely group B and group M. The largest group has 16 members (group S). Throughout the evolutionary tree, the bZIP genes of the three species were distributed in almost all of these 13 subgroups, indicating that the bZIP genes showed different divergence in gene function in pomegranate, *Eucalyptus megacephalus*, and *Arabidopsis*. Meanwhile, some bZIP genes of pomegranate, *Eucalyptus megacephalus* and *Arabidopsis* each clustered together in a small clade, suggesting that a co-speciation event and species-specific duplication events occurred during the bZIP family divergence. Similar to the evolutionary relationships in *Arabidopsis*, our further analysis revealed that two pairs of homologous genes, PgbZIP16/AtHY5 and PgbZIP34/AtHYH, in group H, were able to influence anthocyanin accumulation.

### Gene structure and protein conserved motifs of PgbZIP genes family

**Fig. 3:**
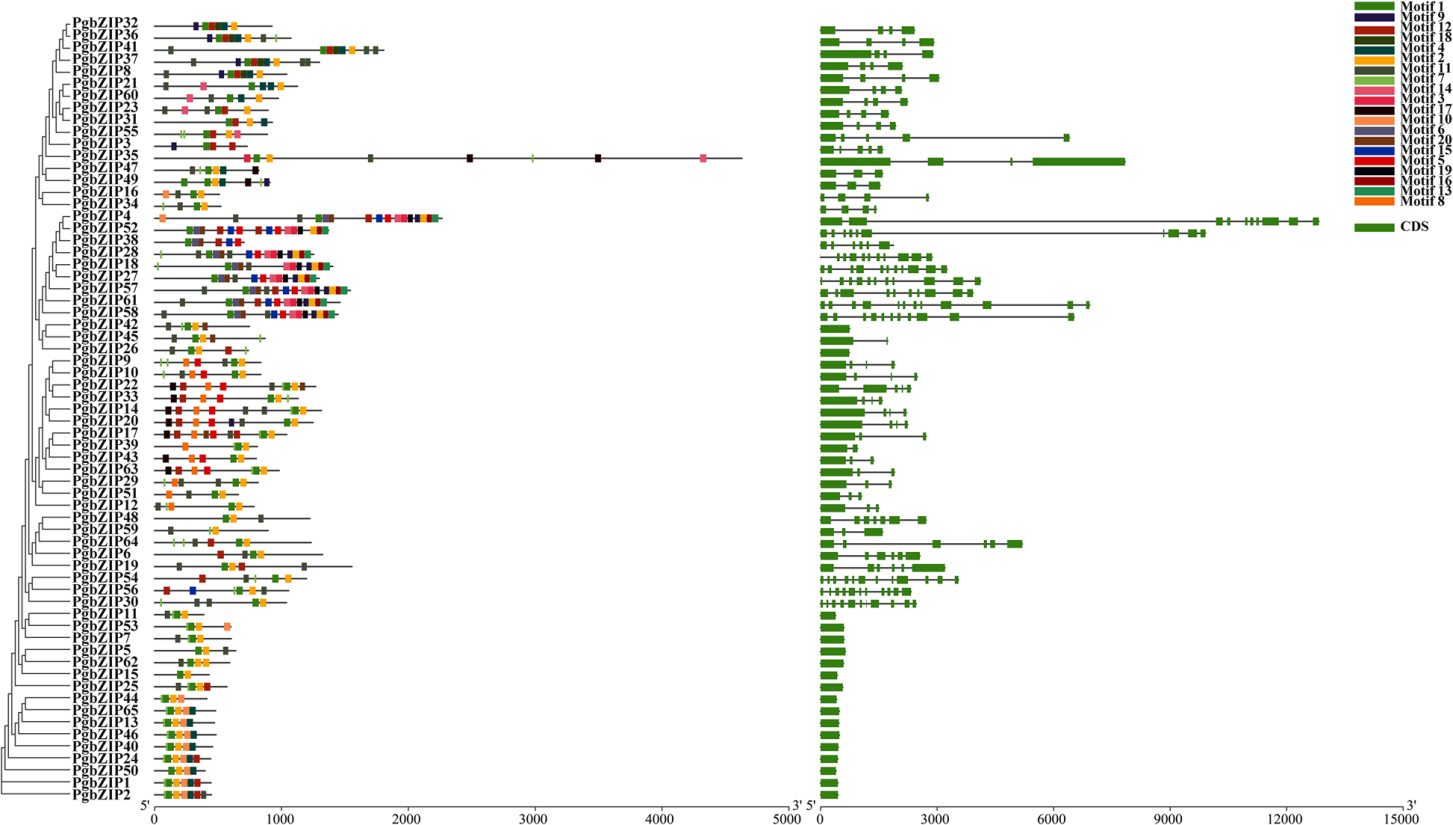
Phylogenetic tree, conserved motifs and gene structures of the PgbZIP gene family. Gene structures. Exons and Intron are displayed using Black lines and green bars. Protein motifs in the bZIP members. The colorful boxes delineate different motifs. The clustering is performed according to the results of phylogenetic analysis

As the composition of introns/exons and types and numbers of introns were typical marks of evolution within certain gene families, we explored the gene structures of PgbZIP genes structures to further understand their evolutionary trajectory (Fig. 3). We analyzed the intron/exon and motif structure of each member. As expected, members of the different groups had different gene structures, conserved domains and numbers of introns/exons, with the number of introns ranging from 0 to 11. For example, there were no introns in the S group, while *PgbZIP54* and *PgbZIP61* had the largest number of introns with 11 introns.

To investigate the distribution of conserved patterns bZIP proteins, 65 PgbZIP protein sequences were analyzed by MEME (Fig. 3). The number and type of conserved motifs contained in each protein sequence varied. The distribution of different conserved motifs was revealed with different functions of different genes. Group D has the most motifs, with PgbZIP4 containing 16 motifs, and Group S has the least motifs, with PgbZIP15 containing 2 motifs.

### Cis-acting elements of pomegranate bZIP gene family

**Fig. 4:**
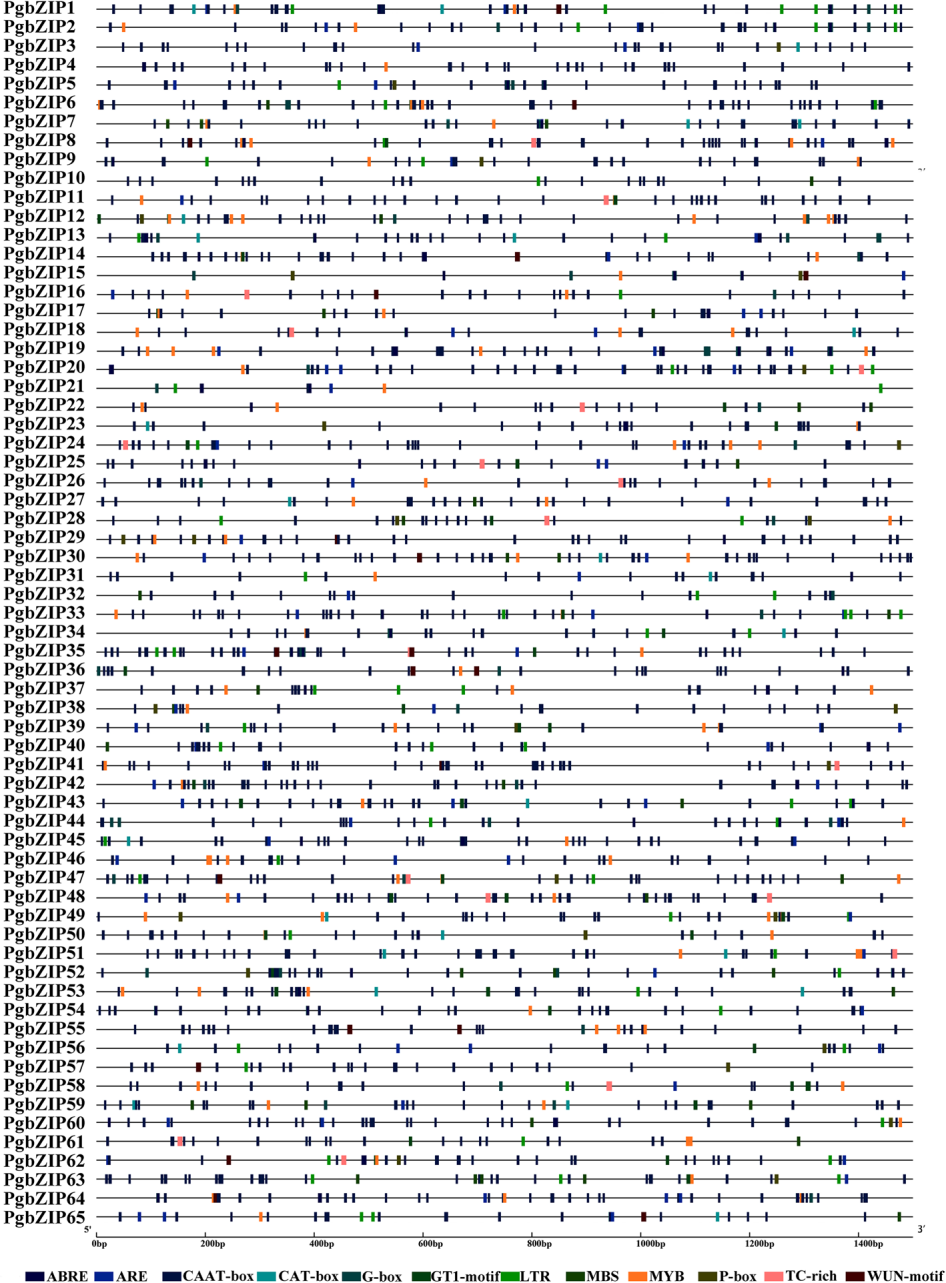
The distribution of cis-acting elements in the promoter region of pomegranate bZIP gene

To further explore the potential mechanism of bZIP gene in biotic and abiotic stress, we submitted the 1500 bp upstream sequence of the PgbZIP translation start site to Plant CARE for detection of cis-acting elements. The PgbZIP gene family cis-acting elements were mapped using the online website GSDS2.0 (Fig. 4). Meanwhile, we analyzed and screened 12 cis-acting elements, mainly including ABA-responsive element ABRE, drought-inducible response element MBS, low-temperature response element LTR, defense and stress-responsive element TC-rich repeats, trauma-responsive element WUN-motif, gibberellin-responsive element P-box, anaerobic-inducible cis-regulatory element ARE, meristematic tissue expression-related cis-regulatory element CAT-box, regulatory element MYB of secondary metabolic pathways, and common cis-acting element CAAT-box of promoter and enhancer regions. Pomegranate had 65 PgbZIPs, each with one or more cis-acting elements, suggesting that expression of PgbZIPs may be associated with these abiotic stresses. In total, 65 genes had one or more ABA response elements and 40 PgbZIPs had one or more LTR response elements, indicating that PgbZIPs may be significantly responsive to ABA and low temperature stresses. 18 PgbZIPs had TC-rich repeats response elements and 17 PgbZIPs had WUN-motif response elements. In conclusion, the analysis of cis-acting elements suggested that the PgbZIP genes may respond to different abiotic stresses.

### Tissue-differential gene expression patterns of pomegranate *bZIP* genes

**Fig. 5:**
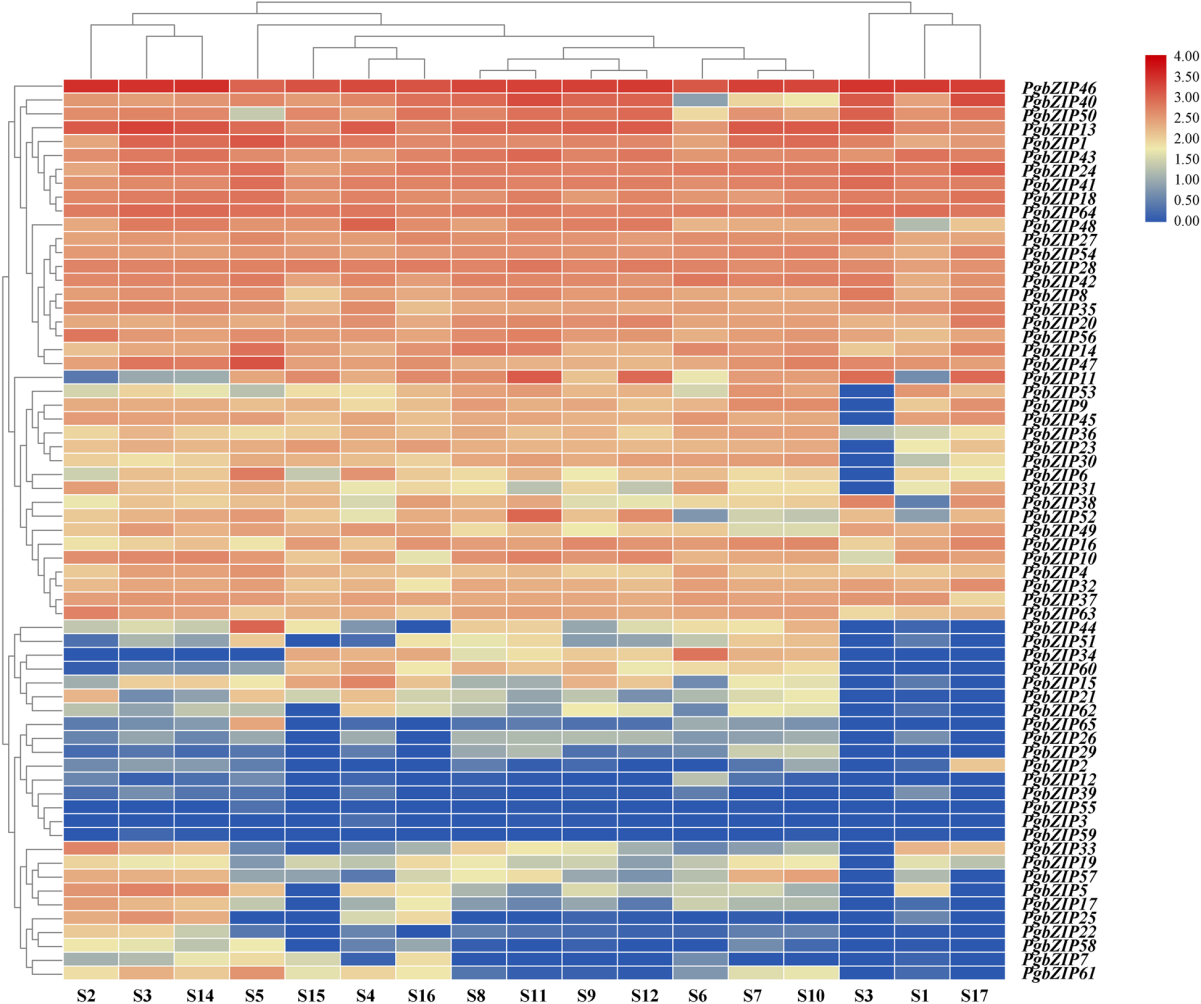
Heatmap of *PgbZIP* gene expression in *P. granatum.* Note: S1: Outer seed coat; S2: Inner seed coat; S3: Pericarp; S4: Flower; S5: Root; S6: Flesh leaf; S7: Bisexual flowers (3.0-5.0 mm); S8: Bisexual flowers (5.1-13.0 mm); S9: Bisexual flowers (13.1-25.0 mm); S10: Functional male flowers (3.0-5.0 mm); S11: Functional male flowers (5.1-13.0 mm); S12: Functional male flowers (13.1-25.0 mm); S13: Inner seed coat of ‘Tunisia’; S14: Inner seed coat of ‘Baiyushizi’; S15: Mix of leaves, flowers, fruit and roots of ‘Black127’; S16: Mix of leaves, flowers, fruit and roots of ‘nana’; S17: Peels of ‘Wonderful’ (cultivars S1-S6 are ‘Dabenzi’, cultivars S7-S13 are ‘Tunisia’)

To explore expression patterns of the pomegranate bZIPs gene family in different tissues, we analyzed the expression of *bZIP* genes in pomegranate roots, stems, flowers, endocarp, exocarp and leaves using transcriptome analysis data by RNA-Seq. As shown in Fig. 5, there was a clear tissue-organ specificity in the expression pattern of *PgbZIP* genes. the expression of *PgbZIP1*, *PgbZIP13*, *PgbZIP18*, *PgbZIP24*, *PgbZIP41*, *PgbZIP43*, *PgbZIP46* and *PgbZIP64* was higher than other members, indicating that this gene may be involved in the transcriptional regulation of various physiological and biochemical processes during pomegranate development. In contrast, the expression of *PgbZIP3*, *PgbZIP29*, *PgbZIP55* and *PgbZIP59* was generally lower. Compared with other tissues, *PgbZIP44* and *PgbZIP47* were highly expressed in roots, *PgbZIP11* in buds and pericarp, and *PgbZIP34* and *PgbZIP46* in young leaves. *PgbZIP16* and *PgbZIP34* were somewhat expressed in pericarp, leaves, flowers and fruits, but relatively low in root development.

### Cloning and analysis of *PgbZIP16* and *PgbZIP34*

**Fig. 6:**
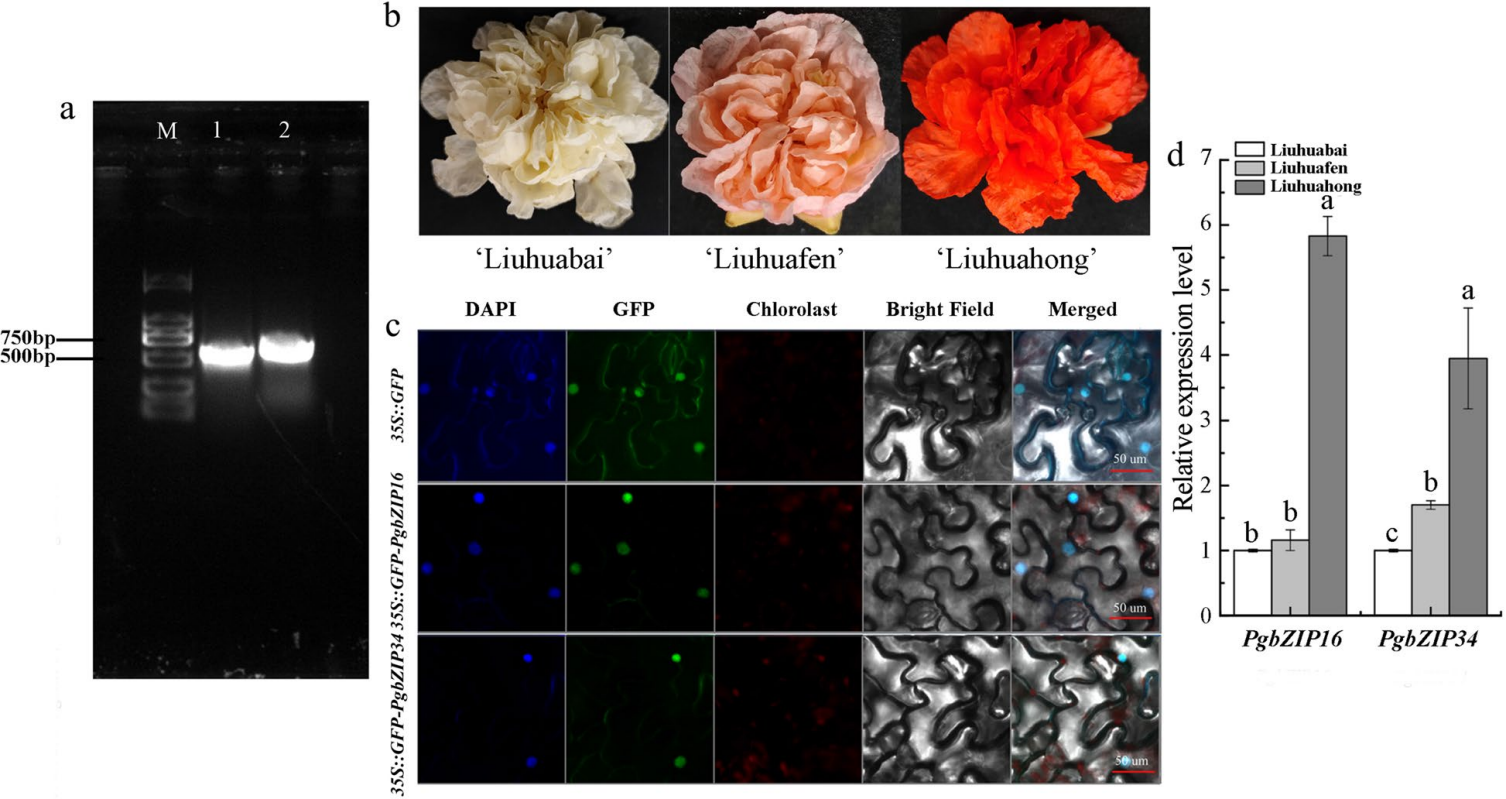
Cloning and characteristics of *PgbZIP16* and *PgbZIP34*. **a**. PCR amplification of *PgbZIP16* and *PgbZIP34*. M: DL ladder 2000 DNA Marker. 1: PgbZIP16. 2: PgbZIP34. **b**. Three different colors of pomegranate flowers. **c**. Subcellular localization of the 35S::GFP-PgbZIP16 and 35S::GFP-PgbZIP34 fusion protein in tobacco leaves. Free GFP served as a control. A DAPI staining assay was conducted to confirm the nuclear localization. Bars = 50 μm. **d**. Tissue petal stage-specific expression of *PgbZIP16* and *PgbZIP34* in pomegranate. Different lowercase letters indicate significant differences at the 0.05 level

The 510 bp and 522 bp open reading frame (ORF) of the *PgbZIP16* and *PgbZIP34* genes were amplified from the mixed-sample cDNA (Fig. 6A). The ORF encodes 170 and 174 aa, respectively. The predicted protein molecular weights were 39,591.74 and 41,604.78 Da, and the theoretical isoelectric points were 5.22 and 5.18, respectively. The amino acid sequence analysis of the proteins of these two genes contains a bZIP domain (BRLZ Domain) located at sites 87 ~ 151 and 96 ~ 160, respectively. Evolutionary tree analysis showed that *PgbZIP16* and *PgbZIP34* genes belong to *HY5* and *HYH* type transcription factors in the bZIP gene family, respectively, and play an important role in the transcriptional regulation of anthocyanin synthesis. Therefore, *PgbZIP16* and *PgbZIP34* were selected for cloning and functional analysis in this paper.

Reverse transcription quantitative PCR (RT-qPCR) revealed that *PgbZIP16* and *PgbZIP34* were highly expressed in ‘Liuhuahong’ (Fig. 6C). Among the three different colors of pomegranate flowers, the expression level *PgbZIP16* in ‘Liuhuahong’ was 5.83 times that of ‘Liuhuabai’ and 5 times that of ‘Liuhuafen’. *PgbZIP34* had a similar expression trend, and its expression level was 3.9 times that of ‘Liuhuabai’ and 2.3 times that of ‘Liuhuafen’.

To determine the subcellular localization of PgbZIP16 and PgbZIP34, the construct encoding PgbZIP16 and PgbZIP34 fused to green fluorescent protein (GFP) were transformed into tobacco leaves. Intense fluorescence from 35S::GFP-PgbZIP16 and 35S::GFP-PgbZIP34 were detected in the nucleus (Fig. 6D), indicating that PgbZIP16 and PgbZIP34 localize to nucleus. These results suggested that PgbZIP16 and PgbZIP34 might function as a transcription factor in regulating anthocyanin biosynthesis.

### Functional studies in tobacco

**Fig. 7:**
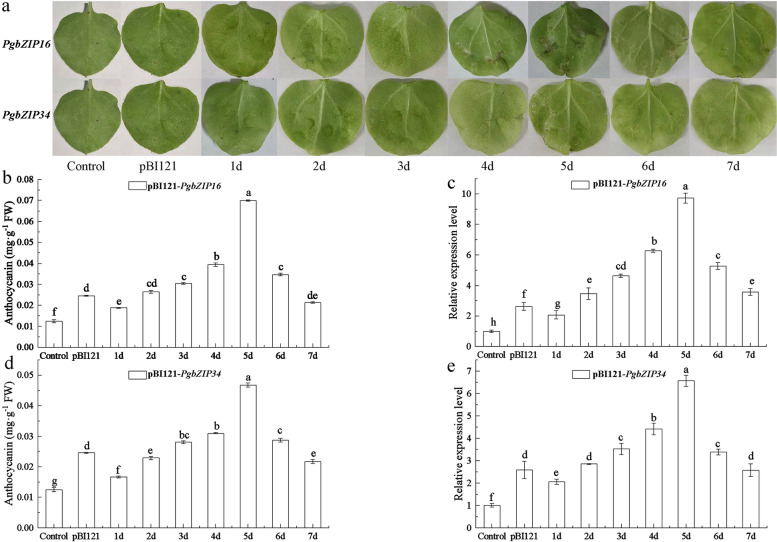
Transient expression of *PgbZIP16* and *PgbZIP34* in tobacco. **a**. Images of wild-type, pBI121-overexpressing, *PgbZIP16*-overexpressing, and *PgbZIP34*-overexpressing tobacco leaves. **b**. Anthocyanin content in wild-type and *PgbZIP16*-overexpressing leaves. **c**. Relative expression level in wild-type and *PgbZIP16*-overexpressing leaves. **d**. Anthocyanin content in wild-type and *PgbZIP34*-overexpressing leaves. **e**. Relative expression level in wild-type and *PgbZIP34*-overexpressing leaves. Different letters indicate significant at the *P* < 0.05 level

To investigate the functions of *PgbZIP16* and *PgbZIP34* genes, we constructed pBI121-*PgbZIP16* and pBI121-*PgbZIP34* overexpression vectors and transferred them into tobacco leaves by injection method. The results showed (Fig. 7a) consistent trends were observed in anthocyanin accumulation and gene expression levels, both of which increased and then decreased. The anthocyanin in *PgbZIP16* transgenic tobacco leaves started to increase significantly on the 3th day, and reached the highest level on the 5th day, which was 0.067 mg·g^−1^ FW. It was 5.58 times higher than that in non-infested leaves and 2.79 times higher than that in pBI121 null leaves. Gene expression was consistent with the level of anthocyanin content, with the highest at day 5, which was 9.72-fold higher than that of un-infested leaves and 3.74-fold higher than that of pBI121 null leaves (Fig. 7b-c). The anthocyanin in *PgbZIP34* transgenic tobacco leaves started to increase significantly on the 3th day, and reached the highest level on the 5th day, which was 0.047 mg·g^−1^ FW. it was 3.92-fold higher than that of un-infested leaves and 1.96 times more than that of pBI121 nulled leaves. Gene expression was consistent with anthocyanin content levels, with the highest being on day 5, which was 6.56 times higher than that of un-infested leaves and 2.54 times higher than that of pBI121 unloaded leaves (Fig. 7d-e). In summary, *PgbZIP16* had an important role in anthocyanin accumulation, but because they are homologous sequences. Therefore, we further explored the genetic regulation of anthocyanin accumulation by *PgbZIP16* in *Arabidopsis*.

### Genetic regulation of *PgbZIP16* on Arabidopsis anthocyanin accumulation

**Fig. 8:**
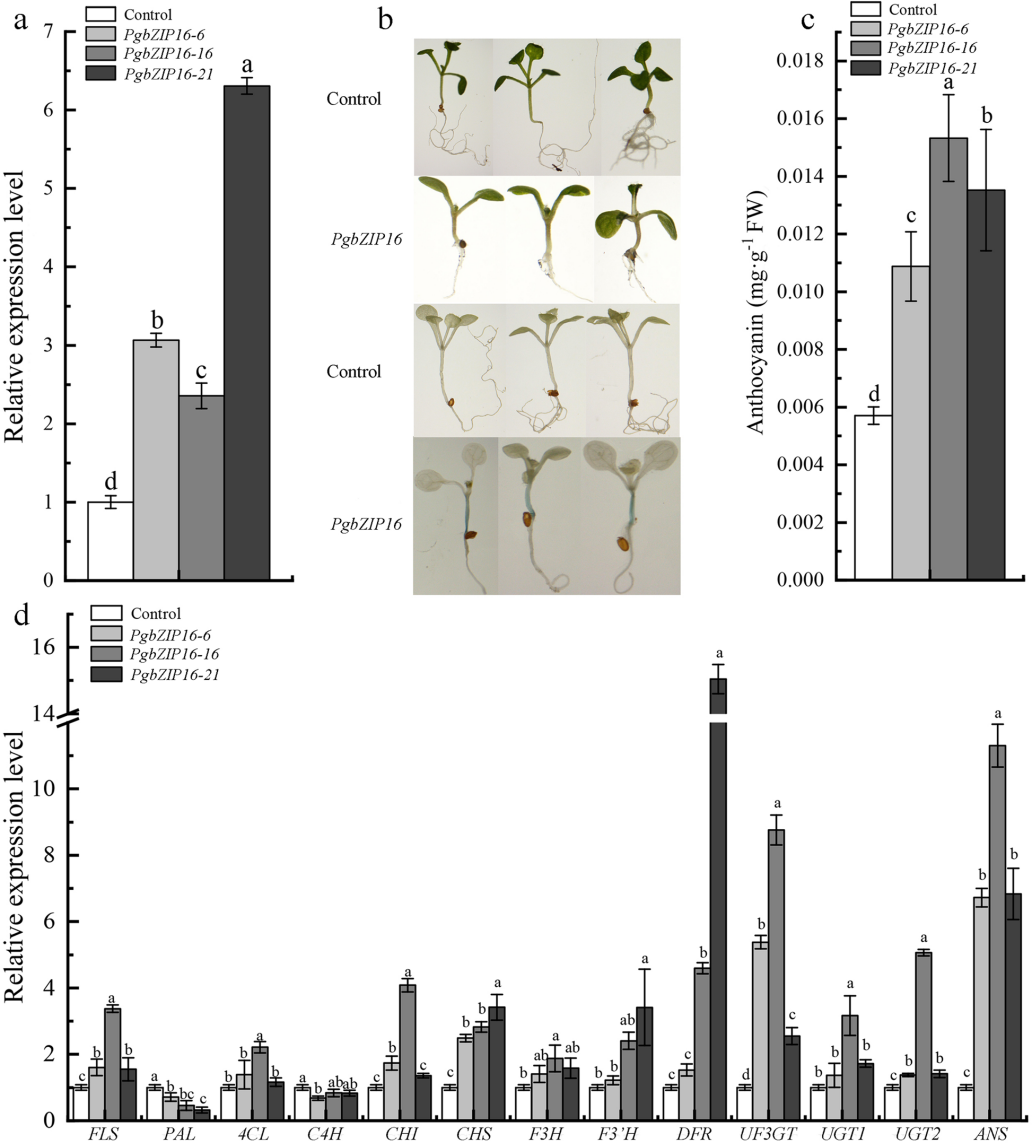
Genetic regulation of *PgbZIP16* on Arabidopsis anthocyanin accumulation. **a**. Expression analysis of *PgbZIP16* in the wild-type, *PgbZIP16* overexpressing *Arabidopsis*. **b**. Images of representative seedling of wild-type, *PgbZIP16* overexpressing lines. **c**. Overexpression of *PgbZIP16* facilitated anthocyanin accumulation in *Arabidopsis*. **d**. Expression levels of anthocyanin-related genes in wild-type, *PgbZIP16* overexpressing *Arabidopsis*. Total RNA extracted from the different genotypic leaves. The different letters indicate statistically significant differences compared with the corresponding wild-type (*P* < 0.05)

It has been previously reported that *HY5* acted as a transcriptional activator that positively regulated the biosynthesis of anthocyanins in plants [38–40]. *HY5* regulated the accumulation of anthocyanins through directly binding to the promoters of *CHS*, *CHI*, *F3H*, *F3′H*, *DFR*, and *ANS* [49, 50]. To investigate whether *PgbZIP16* plays a role in promoting anthocyanin biosynthesis in *Arabidopsis*, we constructed a pBI121-*PgbZIP16* overexpression vector and transformed *Arabidopsis* using the flower-dip method. The *PgbZIP16* overexpression *Arabidopsis* (*PgbZIP16*-6, *PgbZIP16*-16, *PgbZIP16*-21) were compared with control *Arabidopsis* carrying only the empty vector of the 35S:pBI121 vector. It was confirmed that *Arabidopsis* overexpressing *PgbZIP16* expressed *PgbZIP16* at significantly higher levels than control *Arabidopsis* (Fig. 8a). The *PgbZIP16* was constructed into the pBI121 vector to drive stable expression of the GUS reporter gene. Histochemical staining for GUS activity showed that *Arabidopsis* seedlings overexpressing *PgbZIP16* were expressed in stem segments (Fig. 8b). Anthocyanin content was measured in control and *PgbZIP16* overexpressing *Arabidopsis* leaves and was found to be significantly higher in the leaves of *PgbZIP16* overexpressing *Arabidopsis* than in the control (Fig. 8c). In transgenic *Arabidopsis*, there was a concordance between the expression of most structural genes on the anthocyanin biosynthetic pathway (*FLS*, *4CL*, *CHI*, *CHS*, *F3H*, *F3’H*, *DFR*, *UF3GT*, *UGT1*, *UGT2*, and *ANS*) and *PgbZIP16* genes, which were significantly up-regulated compared with the control (Fig. 8d). Moreover, *PgbZIP16* significantly up-regulated the expression levels of *UF3GT*, *ANS*, and *DFR* genes in transgenic *Arabidopsis* and enhanced anthocyanin accumulation. Therefore, the *PgbZIP16* gene has a genetic regulatory effect on anthocyanin accumulation in *Arabidopsis*.

## Discussion

The bZIP transcription factor plays a crucial role in plant growth, development, and abiotic stress responses, such as seed maturation, flower development, stress response, ect [25, 26, 33]. Currently, the bZIP gene family has been studied in the model plant, especially for *Arabidopsis* and crops, such as *AtbZIP11/18* in *Arabidopsis* [27, 29], *GsbZIP67* in soybeans [32], *CabZIP25* in pepper [33], *TabZIP15* in wheat [34] and *MdHY5*/*MdbZIP44* in apple [40, 43]. Until now, despite the sequencing of the whole pomegranate gene has been completed, little is known about the bZIP gene family in pomegranate [45, 47, 48]. So, we identified and analyzed the expression pattern of bZIP based on phylogenetic analysis to speculate on the evolution of the PgbZIP gene family.

In this study, a total of 65 PgbZIP genes were identified in pomegranate using bioinformatics methods (Table. 1). The bZIP members in pomegranate were similar to those in *Arabidopsis* [18], apple [22], poplar [23] and jujube [24], which may be caused by ancient polyploid events. To further understand the evolutionary relationships between *Arabidopsis* and pomegranate, we constructed a phylogenetic tree of bZIP genes following a clustering approach (Fig. 2) [18]. Due to the highly conserved bZIPs sequences, genes with the same function belong to the same group, which provides a reference for studying this gene family. Similar to the *Arabidopsis* grouping, the pomegranate bZIP genes were divided into 13 groups.

Gene structure and conservative motifs were also important basis for studying gene evolution and gene duplication (Fig. 3). We analyzed in detail the structure of the pomegranate bZIP gene and the number of introns and exons. Compared with other gene families, we found that the pomegranate bZIP gene structure was relatively simple, with the number of introns ranging from 0 to 11. In conclusion, most of the PgbZIP genes have similar number of introns compared to other plant species [21, 51–53]. In *Arabidopsis*, the subfamily-specific and conserved motifs may play important roles in the functional differentiation of AtbZIPs subfamilies. For example, most members of group A participate in ABA biological pathway and regulate plant responses to abiotic stress [54–56]. Therefore, the PgbZIP genes in group A could have similar functions. The significant feature of group C members was the extension of the leucine zipper region, which can be up to 9 repeats. In addition, potential target sites for protein modification, such as phosphorylation sites that regulated nuclear translocation and DNA binding, were also preserved [17]. Group D genes could participate in plant defense against pathogens [17]. Group G gene and their homologues were mainly involved in the signal transduction of blue-violet light [57]. Group H contained two genes that could be directly combined with light-induced gene promoters to regulate plant cell elongation, chloroplast synthesis, hormone synthesis and anthocyanin biosynthesis [58]. Group S had the most members, but the number of well-researched genes was less. Members of this group not only plays an important role in the sucrose metabolism pathway, but also could be activated and transcribed under the antibiotic stress [59]. In our study, transcriptome data indicated that PgbZIPs were highly homologous to *Arabidopsis*, demonstrating similar roles in specific biological processes. It seems that the evolution events in bZIP gene family members have happened before species divergence, which affected their number and function [60, 61].

The expression intensity of pomegranate PgbZIPs in different tissues was further analyzed (Fig. 5). The results showed that the genes were expressed in leaves, roots, flowers, seed coat and envelope, except for *PgbZIP3*, *PgbZIP12*, *PgbZIP39*, *PgbZIP55* and *PgbZIP59*, which were hardly expressed in the tissues. This indicated that the bZIP gene family play an essential role in the growth and development of pomegranate. At the same time, the expression of *PgbZIP46* was found to be higher in all tissues than other genes, especially in roots, leaves, and flowers. This may be that this gene is closely related to growth, development and stress in pomegranate. In addition, we speculated that several genes that were barely expressed in tissues may not be involved in the regulation of pomegranate development or stress.

Anthocyanins are water-soluble pigments involved in pathways of plant secondary metabolism. Anthocyanins were mainly found in flowers, leaves, seed coat and fruits of plants in form of glycoside. Anthocyanins not only provided brilliant colors to plants, but also protected plants from ultraviolet radiation and pathogens [62]. The current research on *HY5* and *HYH* of *Arabidopsis*, tomato and apple is more in-depth. For example, *HY5* and *HYH* in *Arabidopsis* are phytochrome receptors of the light signal pathway downstream. Not only the expression of *EBGs* and *LBGs* was directly activate, *HY5* can positively regulate the transcriptional activation of *AtPAP1* [26, 50, 63]. In apple, bZIP transcription factor gene *MdHY5* could directly promote the expression of *MdMYB10* and *MdMYB1* genes, and positively regulate anthocyanin accumulation by enhancing the interaction with its downstream target genes [31, 32, 40, 43]. In addition, *SlHY5* gene silencing down-regulated the accumulation of anthocyanins in tomato [41]. The phylogenetic analyses demonstrate that *PgbZIP16* and *PgbZIP34* shared higher homology with *AtHY5* and *AtHYH*. Therefore, we speculated that *PgbZIP16* and *PgbZIP34* genes in pomegranate also had similar functions to *Arabidopsis*, tomato and apple.

Pomegranate, as an ancient fruit widely consumed fresh fruit, is an economically important fruit tree crop in China. Preliminary research has been conducted on the coloring mechanism of pomegranate peel, but the mechanism of flower color formation has not been studied in depth. In this study, we performed gene cloning, subcellular localization and functional verification of *PgbZIP16* and *PgbZIP34* in the flower color formation mechanism of three kinds of ornamental pomegranate. We results suggested that the patterns of expression of both genes in red were significantly higher than those in white and pink, this result was consistent with the results of grape hyacinth and red pear (Fig. 6) [64, 65]. To investigate the functions of *PgbZIP16* and *PgbZIP34*, we constructed pBI121-*PgbZIP16* and pBI121-*PgbZIP34* overexpression vectors and transformed tobacco leaves. The results showed that there was a consistency between anthocyanin content and gene expression, which both increased and then decreased (Fig. 7b-e). Both *PgbZIP16* and *PgbZIP34* promoted anthocyanin accumulation in tobacco leaves. Compared with *PgbZIP34*, *PgbZIP16* played a more important role in anthocyanin accumulation. To determine the genetic relationship between *PgbZIP16* and structural genes, we used the dipstick method to transfer the constructed overexpression vector into *Arabidopsis* and obtained *PgbZIP16* overexpression strains. Histochemical staining for GUS activity showed that seedlings overexpressing *PgbZIP16* were specifically expressed in stem segments (Fig. 8b). Such an expression pattern suggested that *PgbZIP16* may be involved in the accumulation of anthocyanins at an early stages of *Arabidopsis* development. Furthermore, further studies shown that the overexpression of *PgbZIP16* significantly promoted the anthocyanin accumulation in the transgenic strain (Fig. 8c). Meanwhile, most genes on the anthocyanin synthesis pathway (*FLS*, *4CL*, *CHI*, *CHS*, *F3H*, *F3’H*, *DFR*, *UF3GT*, *UGT1*, *UGT2*, and *ANS*) and *PgbZIP16* gene expression were consistent (Fig. 8d). The expression of *PAL* and *C4H* was lower in *PgbZIP16* overexpressing plants than in the control, which may result from the fact that *PAL* and *C4H*, as structural genes of the mangiferin synthesis pathway, were not directly involved in anthocyanin synthesis [38]. Based on the present experimental study, further investigation of the relationship between *PgbZIP16* and other transcription factors and their role in the process of flower color formation is the focus of future work.

## Conclusions

In this study, a total of 65 PgbZIP genes were identified in pomegranate using bioinformatics methods, and their bZIP structural domain were determined. We constructed a phylogenetic tree of pomegranate and *Arabidopsis*, and divided the *PgbZIP* genes into 13 groups. Due to the high conservation of bZIP genes, proteins with similar functions were clustered into one group, which provided a reliable basis for studying the functions of related genes in gene families in plants. In addition, we identified two candidate genes in the anthocyanin biosynthesis using transcriptome data analysis and performed their gene cloning, subcellular localization, quantitative fluorescence analysis, transient expression and Arabidopsis transformation. Our results indicated that *PgbZIP16* and *PgbZIP34* had similar regulatory mechanisms in anthocyanin accumulation. It is believed that future studies will elucidate the exact molecular mechanisms by which *PgbZIP16* interacts with other transcription factors to promote anthocyanin accumulation.

## Materials and methods

### Identification and characterization of bZIP gene family members of pomegranate

Sequences with E-value < e^−5^ were identified by HMMER v3.2.1 software based on the hidden Markov models (HMM) profile of the bZIP gene family domain (PF00170) downloaded from the Pfam database (http://pfam.xfam.org/). We used the hmmsearch (http://www.hmmer.org/) with bZIP to search the ‘Taishanhong’ pomegranate amino acid sequences, with a threshold of E-value≤1e^−5^, and manually removed redundancy [66, 67]. At the same time, the bZIP proteins of other species were downloaded from Plant Transcription Factor Database (http://planttfdb.gao-lab.org/index.php) as seed files [68]. Sequence similarity searches to genes in the whole genome sequence of pomegranate were conducted using the BLASTp program on a local NCBI database (E-value <1e^−10^, identity >50%), and removed duplicates.

Combing the comparison results of HMMER and BlastP, using the online software SMART (http://smart.embl-heidelberg.de/), CDD (https://www.ncbi.nlm.nih.gov/cdd) and pfam (http://pfam.xfam.org/) databases were used to confirm the integrity of the conserved bZIP domains, and the sequences that did not contain bZIP conserved structural domains were removed [69, 70]. Isoelectric point (PI), molecular weight (MV) and instability indices of the identified bZIP protein were obtained using the online software ExPaSy-Protparam (https://web.expasy.org/protparam/). Subcellular localization of PgbZIPs was predicted by Cell-PLoc-2 (http://www.csbio.sjtu.edu.cn/bioinf/Cell-PLoc-2/) [71].

### Phylogenetic analysis

To explore the phylogenetic relationships of the pomegranate bZIP gene family, all of the *Arabidopsis* bZIP protein sequences were obtained from TAIR database (https://www.arabidopsis.org/). The amino acid sequences of pomegranate and *Arabidopsis* bZIPs were imported into MEGA 7.0 and multiple sequence comparisons were performed using MUSCLE [18]. Thereafter, we used the maximum likelihood method of MEGA 7.0 to construct phylogenetic trees [72, 73]. The classification of the pomegranate bZIP protein family was referenced from previous studies in Arabidopsis. Finally, the phylogenetic tree was visualized using the EvolView website (https://www.evolgenius.info/evolview/) [74].

### Gene structure and conservative motif analysis

Information on 65 PgbZIPs was obtained from the genome annotation GFF files, and the gene structure of PgbZIP gene family was analyzed by the online Gene Structure Display Serve (GSDS, http://gsds.cbi.pku.edu.cn/). We used ClustalW and WebLogo for multiple sequence comparison and visualization analysis, respectively [75, 76]. Finally, the conserved patterns of the bZIP gene family were identified through the online website MEME (http://meme-suite.org/) [77].

### Analysis of cis-acting elements of pomegranate bZIP gene family

The 1.5 kb promoter sequence upstream of the transcription start site of each PgbZIP gene was extracted from the pomegranate genome sequence and predicted by PlantCARE for cis-regulatory elements (http://bioinformatics.psb.ugent.be/webtools/plantcare/html/) [78]. The results were manually deleted and saved as a file in bed format, and the file was submitted to the online website GSDS 2.0 for visualization.

### Expression analysis of pomegranate bZIP gene family

To study the expression of PgbZIPs in different tissues and organs, we used the NCBI database (http://www.ncbi.nlm.nih.gov/) illumina sequencing platform to obtain 7 tissue transcript datas of pomegranate hermaphrodite, functional male flower, leaf, root, endocarp, ectocarp and pericarp. Their accession numbers were ‘Dabenzi’ SRR5279388, SRR5279391, SRR5279394-SRR5279397; ‘Tunisia’ SRR5446592, SRR5446595, SRR5446598, SRR5446601, SRR5446604, SRR5446607 and SRR5678820; ‘Baiyushizi’ SRR5678819, ‘Black127’ SRR1054190, ‘nana’ SRR1055290 and ‘Wonderful’ SRR080723. The RNA-Seq were quality filtered using the fastp software [79]. To quantify annotated transcript abundance, we used Kallisto version 0.44.0 to obtain transcriptome data, and the transformed TPM value log_2_^(TPM + 1)^ were visualized using the TBtools.

### Experiment material

Research conducted at Baima Base for Teaching and Scientific Research of Nanjing Forestry University, the test materials were ‘Liuhuahong’, ‘Liuhuafen’ and ‘Liuhuabai’white pomegranate. No permission is required for sample collection. Pomegranate samples were collected in June 2020, and samples were frozen in liquid nitrogen and stored in a − 80 °C refrigerator for backup.

### Gene cloning and subcellular localization

Using BioTeke Plant Total RNA Extraction Kit (spin column type) to extract RNA from three flower colors of pomegranate petals. cDNA was obtained using reverse transcription kit (PrimeScriptTM RT reagent Kit with gDNAEraser, TaKaRa), and stored at −20 °C. Oligo 7.0 software was used to design the primers (Table S1), and the primer sequence was synthesized by Shanghai Bioengineering Co., Ltd.

The PCR reaction system was as follows: 2 × Taq Plus Master Mix: 25 μl; F: 1 μl; R: 1 μl; DNA template: 2 μl; Nuclease-free ddH2O: 21 μl. The PCR reaction procedure was as follows: 95 °C: 3 min; 95 °C: 15 s, 58 °C: 45 s, 72 °C: 1 min, a total of 35 cycles; 72 °C: 5 min; 4 °C: storage. The PCR products were separated by 1% agarose gel electrophoresis, and the gel was cut to recover the target fragments. After recovery, ligation, transformation, and sequencing, the CDS sequences of the pomegranate *PgbZIP16* and *PgbZIP34* genes were finally obtained. ExPaSy-Translate online tool (https://web.expasy.org/translate/) was used to translate it into amino acid sequence.

The correct recombinant plasmid obtained by sequencing was transferred into Agrobacterium GV3101 by freeze-thaw method, and then the tobacco leaves were infected by Agrobacterium tumefaciens-mediated method for 3-4 weeks. The empty vector pBI121 with GFP tag was transformed into Agrobacterium tumefaciens GV3101 in this study. *pBI121-GFP* was used as a control. After 24 h of dark culture and light culture respectively, the fluorescence signal was observed under a confocal microscope and photographed.

### Expression specificity of pomegranate *PgbZIP16* and *PgbZIP34*

Real-time fluorescent quantitative PCR was used to study the expression patterns of *PgbZIP16* and *PgbZIP34* genes in the petals of three different flower colors of pomegranate. The BioEasy Master Mix Plus (SYBR Green) was used as a fluorescent dye, and the reaction program was as follows: 95 °C: 3 min; followed by 40 cycles of 95 °C for 30 s and 60 °C for 15 s. Specific primers were designed for qPCR (Table S1). The pomegranate actin was used as the internal reference gene, and was performed on 3 biological and 3 technical replicates for each treatment. Comparison of relative gene expression data of flowers was done using the 2^-ΔΔCt^ method [80, 81].

### Agrobacterium infiltration

*PgbZIP16* and *PgbZIP34* recombinant plasmids with GUS tags were transferred into Agrobacterium tumefaciens GV3101, and the activated Agrobacterium tumefaciens was inoculated into 50 mL of LB liquid medium at a ratio of 1:100, and incubated at 28 °C for 16 h with shaking at 210 r/min. Then centrifuged at 4000 rpm/min for 10 min to collect the bacteria and the bacteria were resuspended and used in permeate (10 mmol/L MES + 10 mmol/L MgCl2.6H2O + 100 mmol/L AS, pH 5.6) to resuspend the bacteria. Then the injection solution was prepared proportionally, placed at room temperature for 2-4 h, injected into the abaxial surface of tobacco leaves, sampled daily after infestation, stored in a refrigerator at −80 °C, and tested 7 days later.

### Overexpression of *PgbZIP16* in *Arabidopsis* and GUS activity assay

Wild-type *Arabidopsis* plants were grown in the incubator (*Arabidopsis* seeds were kept in our laboratory). We constructed the pBI121-*PgbZIP16* overexpression vector and transformed it into Agrobacterium GV3101, inoculated it in 50 mL LB liquid medium, incubated at 210 r/min at 28 °C for 48 h, and then collected the bacteria by centrifugation at 4000 rpm/min within 10 min. After re-suspension of bacteria with an osmotic agent (0.05% sliwet77 + 5% sucrose +1/2MS liquid medium). *Arabidopsis* plants were subsequently transformed according to the flower dip method and incubated in the dark for 48 h. After three infestations, *Arabidopsis* seeds were collected and screened for *PgbZIP16* transgene-positive plants [82].

Seeds of the identified positive plants were planted in 1/2 MS Petri dishes containing 25 μg/L. Positive seedlings with two true leaves were photographed after two weeks, and selected plants were selected and placed in prepared GUS staining solution and incubated overnight at 37 °C. Positive stained plants were decolorized with anhydrous ethanol and photographed with a stereomicroscope after all the green color faded [83].

### Statistical analysis

The data are shown as the means ± standard errors (SEs) of 3 or 6 independent biological replicates. Statistical differences between samples were analyzed by LSD and Duncan (D) (*p* < 0.05). Data analysis and visualization were processed using SPSS 20.0 and Origin 2018.

## Supplementary Information

**Additional file 1: Supplementary Table 1**. Primers for the gene cloning, subcellular localization and qRT-PCR.

## Data Availability

The whole genome data of pomegranate is downloaded from the NCBI database (https://www.ncbi.nlm.nih.gov/search/all/?term=ASM220158v1), and the accession number is ASM220158v1. The transcriptome data is obtained from NCBI (https://www.ncbi.nlm.nih.gov/Traces/study/), and the accession numbers are SRP103147 and SRP100581. The bZIP protein sequences of *A. thaliana* downloaded from the PlantTFDB database (http://planttfdb.gao-lab.org/index.php). Public access to all databases is open. The datasets supporting the conclusions of this article are included within the article (and its additional files).

## Abbreviations

bZIP: Basic Leucine Zipper
MCScanX: Multiple Collinearity Scan toolkit
pI: Theoretical Isoelectric Point
Mw: Molecular Weight
ML: Maximum Likelihood
cDNA: Complementary DNA
qRT-PCR: Quantitative real-time polymerase chain reaction
