# Supplementary material for: Genome-wide identification and characterization of bZIP gene family and cloning of candidate genes for anthocyanin biosynthesis in pomegranate (Punica granatum)
Source: BMC Plant Biol. 2022 Apr 4;22:170. doi: 10.1186/s12870-022-03560-6 (PMC8978422; doi:10.1186/s12870-022-03560-6)
Supplement: Supplementary file 1 — Additional file 1: Supplementary Table 1. Primers for the gene cloning, subcellular localization and qRT-PCR. [file 12870_2022_3560_MOESM1_ESM.docx]

**Supplementary table 1 Primers for the gene cloning, subcellular localization and qRT-PCR**

| Primer | Primer sequence（5’-3’） | Annotation |
| --- | --- | --- |
| *PgbZIP16* | F: ATGCAAGAGCAGGCGACGA  R: TACAAAGACCCGTCGGCAT | Gene clone |
| *PgbZIP34* | F: ATGTCGTCAGCGGTGCAG  R: TCATTGCTTTGGTTCGGTGT | Gene clone |
| GFP*-PgbZIP16* | F: gagaacacgggggactctagaATGCAAGAGCAGGCGACGA  R: gcccttgctcaccatggatccTACAAAGACCCGTCGGCAT | Subcellular localization |
| GFP*-PgbZIP34* | F: gagaacacgggggactctagaATGTCGTCAGCGGTGCAG  R: gcccttgctcaccatggatccTCATTGCTTTGGTTCGGTGT | Subcellular localization |
| GUS-*PgbZIP16* | F: gagaacacgggggactctagaATGCAAGAGCAGGCGACGA  R: ggactgaccacccggggatccCAAAGACCCGTCGGCAT | Genetically modified verification |
| qRT-*PgbZIP16* | F: AACAGAGTTTCAGCGCAGCA  R: GGCCTTCTTCCGCTCCCT | Gene expression |
| qRT-*PgbZIP34* | F: ACTGCCACGACTAACTCCAC  R: CCATCAGGCCCCATTTGC | Gene expression |
| *PgActin* | F: AGTCCTCTTCCAGCCATCTC  R: CACTGAGCACAATGTTTCCA | Gene expression |
| *4CL* | F: CGCAAACCCTTTCTACACT  R: CAATTCTCCGGTGTTGGTTC | Gene expression |
| *C4H* | F: CCTCCATGATGCGAAGCTC  R: GCTTCGACGTCCAACACC | Gene expression |
| *PAL* | F: GGCCAATTCCGCGACA  R: ATTTAGCTCATCGCGGACA | Gene expression |
| *FLS* | F: AGAGACCAGCACAAGCC  R: GCCACTTTACGCGCCAC | Gene expression |
| *CHS* | F: CGTCTTCTGCACTACCTCC  R: TGGTACATCATGAGACGCTT | Gene expression |
| *CHI* | F: CCATCTCCAAACGCGAAA  R: AGCCAATAAGTTCTCGTCCA | Gene expression |
| *F3H* | F: GTGTTTAGCGACGAAATCCC  R: TCTCACAAGCCTCAACGA | Gene expression |
| *F3’H* | F: ACTAAGCCTCATCGAACCC  R: ATGCCATGTGTTTAGCTCC | Gene expression |
| *DFR* | F: TTGTTCGTGCCACCGTTC  R: TCCATTCACTGTCGGCTT | Gene expression |
| *ANS* | F: TCTCTCTGTCGGTCTAGGTT  R: AGTATCCCCAATGTGCATC | Gene expression |
| *UF3GT* | F: ATGTATCCGTGGTTAGCCTT  R: AGTTTGTCTCCGCACCC | Gene expression |
| *UGT1* | F: TCAACCGACGAACGCGAA  R: CCTCAACTCCACCGACT | Gene expression |
| *UGT2* | F: CCTCTCCTTCCACCGTCT  R: CCGCTAAACACGTATCCCT | Gene expression |
